# Sensors for Structural Health Monitoring and Condition Monitoring

**DOI:** 10.3390/s21051558

**Published:** 2021-02-24

**Authors:** Francesc Pozo, Diego A. Tibaduiza, Yolanda Vidal

**Affiliations:** 1 Control, Modeling, Identification, and Applications (CoDAlab), Department of Mathematics, Escola d’Enginyeria de Barcelona Est (EEBE), Campus Diagonal-Besòs (CDB), Universitat Politècnica de Catalunya (UPC), Eduard Maristany, 16, 08019 Barcelona, Spain; yolanda.vidal@upc.edu; 2 Departamento de Ingeniería Eléctrica y Electrónica, Universidad Nacional de Colombia, Cra 45 No. 26-85, Bogotá 111321, Colombia; dtibaduizab@unal.edu.co

Structural control and health monitoring as condition monitoring are some essential areas that allow for different system parameters to be designed, supervised, controlled, and evaluated during the system’s operation in different processes, such as those used in machinery, structures, and different physical variables in mechanical, chemical, electrical, aeronautical, civil, electronics, mechatronics, and agricultural engineering applications, among others. Continuous monitoring of these structures is a need because these are subject to changes in environmental and operation conditions along their lifetime, which can result in changes and possible fails and damages in all the structure and their components. The proper development of these applications is associated with the use of reliable data from sensors or sensor networks, which requires the use of advanced signal processing techniques, sensor data fusion, and data processing (sometimes in real-time) to produce a reliable system and avoid accidents or failures in the process.

After a rigorous peer review process, a total of 19 papers were published, covering different aspects of condition monitoring, structural control, and health monitoring (SCHM).

Damage identification process is addressed into the structural health monitoring (SHM) task to determine different levels of the state of a structure. These levels include damage detection and localization, the knowledge of the type and extent of damage, the prediction of the remaining lifetime, and the development of smart structures. Each of these steps can be addressed from different points of view, but one of the more used is data-driven strategies. In [1], the use of data-driven algorithms is explored at each level of the damage diagnosis as well as the instrumentation and implementation process to show the current state of some of the developments of data-driven SHM.

In terms of the use of sensors for industrial applications, the design of algorithms and methodologies to process all data from sensors still remains as an open research topic. In the case of the development of artificial taste recognition systems, it implies the use of data-driven algorithms and methodologies to monitor the condition and quality of a process as in the case of the food industry. The work of [2] explores the use of a new nonlinear feature extraction-based approach using manifold learning algorithms to improve the classification accuracy in an electronic tongue sensor array. The paper results show that it is possible to perform the classification in a data set belonging to seven different aqueous matrices with nine samples per class for a total of 63 samples with an accuracy of 96.83%.

During its operation, the structures are subject to conditions that can result in failure or damage and affect its normal behavior. Structures using reinforced concrete can be affected by corrosion resulting in risk for operation. As a contribution to monitoring these kinds of structures, in [3] the authors develop a device that can be embedded into the concrete at various locations and depths to monitor corrosion. Results show that it is possible to determine the corrosion in a structure by measuring its electrical resistance with the developed device.

SHM and condition monitoring have multiple applications in civil infrastructure and multiple works address developments to monitor beams. In [4] it is possible to find an alternative to the techniques that make use of structural analysis and strain gauge measurements to locate the neutral axis of a T-Beam bridge. This work shows the analysis of ultrasonic coda waves in a sensor network and explores its advantages in locating the neutral axis and evaluate the global structural health and inner damages.

In [5], a methodology is described that makes use of a damage index for degradation assessment of concrete beams. This methodology considers two steps starting with the use of raw acoustic emission data from degradation in concrete beams and after the use of the damage index, which considers the use of 5 acoustic emission burst features and a Mahalanobis–Taguchi system to classify the condition of the structure under test.

Applications of condition monitoring are more common in machinery by its multiple advantages. An example of this development can be found in [6] where a dynamic monitoring system to monitor and evaluate the dynamic response of the triad formed by an UVABB tool, milling machine, and C45 steel work-piece is presented. The work is validated by using five accelerometers, and a dynamometric table is proposed and proved to be effective in characterizing the whole system.

Structural health monitoring of civil structures, such as bridges or dams, has received much attention in the last few years, since they are key components of transportation infrastructure [7,8,9]. In [7], a damage detection method is proposed to locate and quantify a damage in a bridge subjected to a moving load. Since recording input excitation in a bridge may be an extremely difficult task, this paper presents an output-only vibration-based approach for the bridge health monitoring. The lack of investigation on the direct use of time-domain filters in the field of structural health monitoring lead the authors to explore the direct application of the Savitzky–Golay filter (SGF) for damage localization and quantification. One of the main advantages of the proposed method is that this approach is insensitive to noise. Furthermore, fitting a Gaussian curve, the proposed method can be viewed as baseline-free.

A different approach for bridge damage identification is presented in [8], with an additional virtual mass for damage identification and where a vehicle bump is considered as the excitation. The proposed approach, validated using a numerical example of a two-span continuous beam, broadens the potential application scope in practical engineering by using additional virtual physical parameters. In addition, the presented strategy is equivalent to the addition of a mass to the bridge for testing purposes, without the risks of overloading the structure.

Klun et al. [9] present a methodology to include non-contact vibration monitoring as part of structural health monitoring of concrete dams with the first application of the laser Doppler vibrometer in non-stationary conditions. An important pre-processing of the data is performed to eliminate pseudo-vibrations and measurement noise that are inherent in the conditions of the dam. The use of this laser technology is proven to be able to complement the more standard monitoring activities on large dams.

Continuous health monitoring of transmission networks is a vital task to prevent sudden failure in power transmission lines. In [10], a vibration-based technique to identify the spatial location of existing damage in the power transmission towers is presented. An important advantage of this approach is the use of a limited number of sensors. To optimize the number of sensors, the authors minimize the non-diagonal entries of the modal assurance criterion (MAC) matrix. To extract the modal parameters, two strategies have been used: the Hilbert–Huang transform and continuous wavelet transform (CWT). The approach was validated on a numerical model and the numerical model was verified through modal testing of the actual tower.

An SHM strategy for detection and classification of structural changes based on two-step data integration (data unfolding and mean-centered group-scaling), data transformation using PCA, and a two-step data reduction combining PCA and t-SNE has been proposed in [11]. Some features of this work worth highlighting are the extension and the adaptation of the *t*-SNE algorithm to the field of SHM as well as the high classification accuracy using collected data from the structure and without the use of complex mathematical models. The method is evaluated using experimental data from an aluminum plate with four piezoelectric transducers (PZTs). Results are illustrated in the frequency domain, and they manifest the high classification accuracy and the strong performance of this method.

A typical SHM system consists of a sensor network, a central data acquisition node, and algorithms. Literature research is extensive in the field of algorithm development. However, the instrumentation, and the sensors in particular, is one of the elements in the field of structural health monitoring and condition monitoring that is sometimes less highlighted. It is worth remembering that various types of parameters are measured by the SHM sensor network in which the strain is crucial for assessment of the structure state. In [12], a miniaturization of a microstrip patch strain sensor is proposed and analyzed. The sensor is designed using a specific patch shape, the Sierpinski curve based fractal geometry. Simulation and experimental analysis for all sensors are carried out where a good convergence between results of simulation and measurements is achieved.

In [13], the problem of crack monitoring of metallic materials is faced. Antenna sensors have been generally employed for this purpose, exploiting the mathematical relationship between the surface crack length of metallic material and the resonant frequency, but the influence of the crack depth on the sensor output and the difference of whether the crack is depth-penetrated remained unexplored. The paper, by means of numerical simulations, reveals that the crack depth has a greater influence on the resonant frequency compared with the crack length. The proposed monitoring approach is experimentally validated to be feasible not only for cracks but also for corrosion pits of metallic materials.

The acoustic emission (AE) method is a very popular and well-developed method for passive structural health monitoring of metallic and composite structures. It has been efficiently used for damage source detection and damage characterization in a large variety of structures such as thin sheet metals. On the other hand, piezoelectric wafer active sensors (PWASs) are lightweight and inexpensive transducers, which recently drew the attention of the AE research community for AE sensing. In [14], an understanding of the fatigue crack growth from AE signals in thin sheet metals recorded using PWAS sensors on the basis of the Lamb wave theory is given. Furthermore, this understanding is used to contribute a predictive analytical model of AE signals (sensed by PWAS sensors during a fatigue crack growth event). The analytical model and the simulated and experimental results showed a close match, which verified the analytical prediction.

It is well known that temperature changes can lead to false diagnoses when using piezoelectric sensors and electromechanical impedance technique (EMI) measurements. In [15] a compensation method is developed to face this problem. It can be applied mainly to structures that present impedance amplitude and frequency shifts with linear dependence of the temperature and frequency. The strategy is tested experimentally in two steel pipes—healthy and damaged—compensating the temperature effect ranging from −40° to 80°, with analysis on the frequency range from 5 to 120 kHz. The simulated and experimental results showed that the studies effectively contribute to the SHM area, mainly to EMI-based techniques.

Structural health monitoring (SHM) techniques can diagnose structural damage and assess the structural safety of civil structures such as bridges. Vibration-based approaches have excellent potential. However, bridges inevitably suffer from actions caused by varying environmental temperatures; furthermore, the aforementioned actions may mask the changes in damage features—for example, the natural frequencies of bridges—caused by structural damage. In [16], a hybrid method is proposed to detect the damage of bridges under environmental temperature changes. On one side, the PCA-based method is applied to deal with the non-principal components; on the other side, the Gaussian mixture method is used to classify all principal components into different clusters, and then the stated detection method is implemented to detect bridge damage for each cluster. In this way, all the damage feature information is saved and used to detect the damage. A numerical example, as well as an actual bridge example, show the effectiveness of the proposed approach.

The work by Kim et al. [17] contributes to the application of real-time structural health monitoring for offshore structures, overcoming the problem of applicability in the field due to increasing calculation costs with increasing structural complexity. In particular, a multiple damage detection method using cosine similarity of the rate of change of natural frequencies is proposed. The process is comprised of three parts: (1) a damage estimation matrix is constructed from a finite element model using modal analysis; (2) the rate of change of natural frequencies from operational modal analysis based on sensor data during operating is normalized and utilized as a normalized warning index (when the index becomes larger than a threshold, the damage reflection vector is generated); (3) cosine similarity between the damage estimation matrix and the damage reflection vector is computed, and finally the most similar damage cases among the vector sets of the estimation matrix are identified in the ranking of similarity. Thus, a damage warning and ranking of the most possible damage cases is provided, facilitating its applicability and usability.

Periodic inspection of the technical condition of a railway transport system is essential for maintaining its high reliability and safety. The increasing accuracy of global navigation satellite system (GNSS) measurements provides new opportunities for developing effective inspection methods and designing track axis adjustment projects. In [18], a measuring platform equipped with at least two GNSS receivers installed above the bogie pivot pins is studied. This approach makes it possible to determine more precisely the track axis, and the base vector can be used for qualitative evaluation of the obtained measurement results. That is, the developed method is capable of identifying incorrect measurement (due to obstacles, for example) results with the Savitzky–Golay filter and it is characterized by the high speed of numeric calculations (use of sparse matrices).

Increasing the length of wind turbine blades, for maximum energy capture, leads to larger loads and forces acting on the blades. In particular, alternate bending due to gravity or nonuniform wind profiles leads to increased loads and imminent fatigue. Therefore, blade monitoring in operation is needed to optimize turbine settings and, consequently, to reduce alternate bending. In [19] a novel approach, by using hierarchical clustering, for continuously monitoring blade bending in the operation of the turbine, is proposed. The stated method is characterized by the following advantages: (1) accelerometers at the blade tip allow for a qualitative assessment of alternate bending at reasonable mounting effort; (2) the sensors can operate wirelessly and self-sufficiently; (3) no properties of the blade such as geometry and material are needed; (4) no environmental and operational parameters of the turbine are needed for evaluation.

As it has been said, structural health monitoring and condition monitoring is a research field that is attracting a lot of attention. One example of this interest is the growing number of papers published in this field as well as the open call-for-papers of Special Issues. The journal *Sensors* runs special issues to create collections of papers on specific topics with the aim of building a community of researchers to discuss the latest innovations and ideas and develop new interactions. Without going any further, more than 35 open Special Issues include *structural health monitoring* as a keyword, 18 of which explicitly include *structural health monitoring* in the Special Issue title. A brief selection of Special Issues could include:

- Interferometric Sensors and Sensing Technologies for Structural Health Monitoring, edited by Giovanni Nico, Stefania Campopiano and Giuseppina Prezioso (submission deadline 31 March 2021);
- Acoustic Emission Sensors for Structural Health Monitoring, edited by Tomoki Shiotani (submission deadline 31 March 2021);
- Vision Based Sensors and Sensing Technologies for Structural Health Monitoring, edited by Mohammad Jahanshahi (submission deadline 30 April 2021);
- Structural Health Monitoring with Ultrasonic Guided-Waves Sensors, edited by Gerardo Aranguren and Josu Etxaniz (submission deadline 30 June 2021);
- Toward Data-Driven Structural Health Monitoring: New Approaches and Sensor-Based Methods, edited by Francesc Pozo and Diego Alexander Tibaduiza Burgos (submission deadline 30 June 2021);
- Structural Health Monitoring and Non-Destructive Testing for Engineering Applications: Advances in Sensor and Technologies, edited by Leandro Maio, Vittorio Memmolo and Marco Laracca (submission deadline 31 August 2021);
- Structural Health Monitoring and Nondestructive Evaluation with Ultrasonic Guided Waves, edited by Clifford Lissenden (submission deadline 31 December 2021).

Finally, we would like to express our gratitude to the anonymous reviewers, who dedicated their time reviewing the submitted papers, and to all authors who contributed, as it was with the work, enthusiasm, and motivation of them that this special issue became a reality. Finally, we hope the reader will find this issue enlightening in the compelling area of condition monitoring and structural health monitoring.
